# Experimental investigations on drag-reduction characteristics of bionic surface with water-trapping microstructures of fish scales

**DOI:** 10.1038/s41598-018-30490-x

**Published:** 2018-08-15

**Authors:** Liyan Wu, Zhibin Jiao, Yuqiu Song, Cuihong Liu, Huan Wang, Yuying Yan

**Affiliations:** 1 College of Engineering, Shenyang Agricultural University, Shenyang, 110161 P.R. China; 2 Faculty of Engineering, University of Nottingham, Nottingham, NG7 2RD UK; 3 Centre for Fluids & Thermal Engineering Research, University of Nottingham Ningbo China, Ningbo, China

## Abstract

Biological surfaces with unique wettability in nature have provided an enormous innovation for scientists and engineers. More specifically, materials possessing various wetting properties have drawn considerable attention owing to their promising application prospects. Recently, great efforts have been concentrated on the researches on wetting-induced drag-reduction materials inspired by biology because of their ability to save energy. In this work, the drag-reduction characteristics of the bionic surface with delicate water-trapping microstructures of fish *Ctenopharyngodon idellus* scales were explored by experimental method. Firstly, the resistance of smooth surface and bionic surface experimental sample at different speeds was carefully tested through the testing system for operation resistance. Then, the contact angle (CA) of fish scale surface was measured by means of the contact angle measuring instrument. It was discovered that the bionic surface created a rewarding drag-reduction effect at a low speed, and the drag-reduction rate significantly displayed a downward trend with the increase in flow speed. Thus, when the rate was 0.66 m/s, the drag-reduction effect was at the optimum level, and the maximum drag reduction rate was 2.805%, which was in concordance with the simulated one. Furthermore, a contact angle (CA) of 11.5° appeared on the fish scale surface, exhibiting fine hydrophilic property. It further manifested the spreading-wetting phenomenon and the higher surface energy for the area of apical of fish scales, which played an important role in drag-reduction performance. This work will have a great potential in the engineering and transportation field.

## Introduction

Saving energy and reducing consumption always draw great attentions among a growing number of scholars. In the field of underwater equipments, including ships, underwater detectors, and submarines, according to theoretical calculations, if the frictional resistance has been reduced by around 10 percent, their speed and navigation would be both improved by around 3.57 percent^1^. Research showed that the surface of an aircraft coated with a transparent plastic that had microscopic texture of the scales of a shark could make surface resistance reduce by up to 8%, saving the fuel about 1.5%^2^. Fortunately, nature has always supplied inspirations to scholars. In the last decades, some researchers have made great efforts to address issues concerning the innovation in technology to be inspired by organisms in nature^3–7^. After millions of years of continuous adaption and evolution, bodies of creatures in nature have formed delicate biological surface structures with drag-reducing characteristics^8–11^. For instance, convex hull structures of the head of the dung beetle^12–14^, the herringbone riblet textures inspired by the bird feathers^15,16^, ridged structures distributed on the mouthpart of the honeybee^17,18^, and the super-hydrophobic surface of the skins of many creatures^19–22^. In addition, the surface structure of many aquatic animals also had excellent drag-reduction characteristic, which had been applied in many fields and showed great drag-reduction effect. For instance, the skin of some sharks was covered by tooth-like micro-scales with longitudinal grooves, which could generate the vortex over the skin’s surface. These vortexes could reduce the burst of turbulent intensity near the wall of grooves. Thus, the structures resulted in water moving easily over the shark skin, exhibiting efficient drag-reduction performance^2,23,24^. (Bio-inspired by the typical model with a low drag surface, high-efficiency swimsuits have been designed with an antibacterial effect^25^). Very recently, inspired by the design of ribbed patterns of shark skin, the effects of periodic sinusoidal riblet surfaces aligned in the flow direction on the evolution of a laminar boundary layer flow has been investigated by means of the numerical analysis method^26^. The results demonstrated that these riblets could decrease the shear stress inside the grooves and the total integrated viscous drag force on the plate in the laminar boundary layer regime. Besides, Domel *et al*. has reported a set of denticle structures inspired by the drag-reduction characteristics of the skin of the sharks by the combination of experimental and simulation methods, achieving simultaneous drag-reduction effect and lifting generation on the aerofoil, which effectively improved the aerodynamic performance^27^. Zhang *et al*. investigated the drag-reduction property of aero engine blade with micro-textures through the experimental and simulated approaches, showing better drag-reduction property in comparision with the un-treated blade^28^. The bionic jet surface, inspired by the jet characteristics of shark-gill slits, was established in order to produce a great drag-reduction performance through the method of changing the fluid friction resistance on the surface^29^. And then, it is well-known that the surfaces of dolphins exhibited self-adaptive characteristics, which could reduce the velocity gradient of the boundary layer, making the wall shear force decrease, to realize great drag-reduction effect^11,30–32^. Also, the flippers of humpback whales had some spherical non-smooth structures, which could reduce the flow resistance of its movement^33–35^. In addition, sector like scales of the carps were covered by micro-papillae with nano-structures, which manifested both great drag-reduction effect and superoleophilicity in air and surperolephobicity^36,37^. For the carassius auratus, the apical of scales was covered by micron-scale indentations with diameter range of 5 um to 10 um, and the results showed that the micron-scale structures had a remarkable drag-reduction effect^38^.

**Figure 1:**
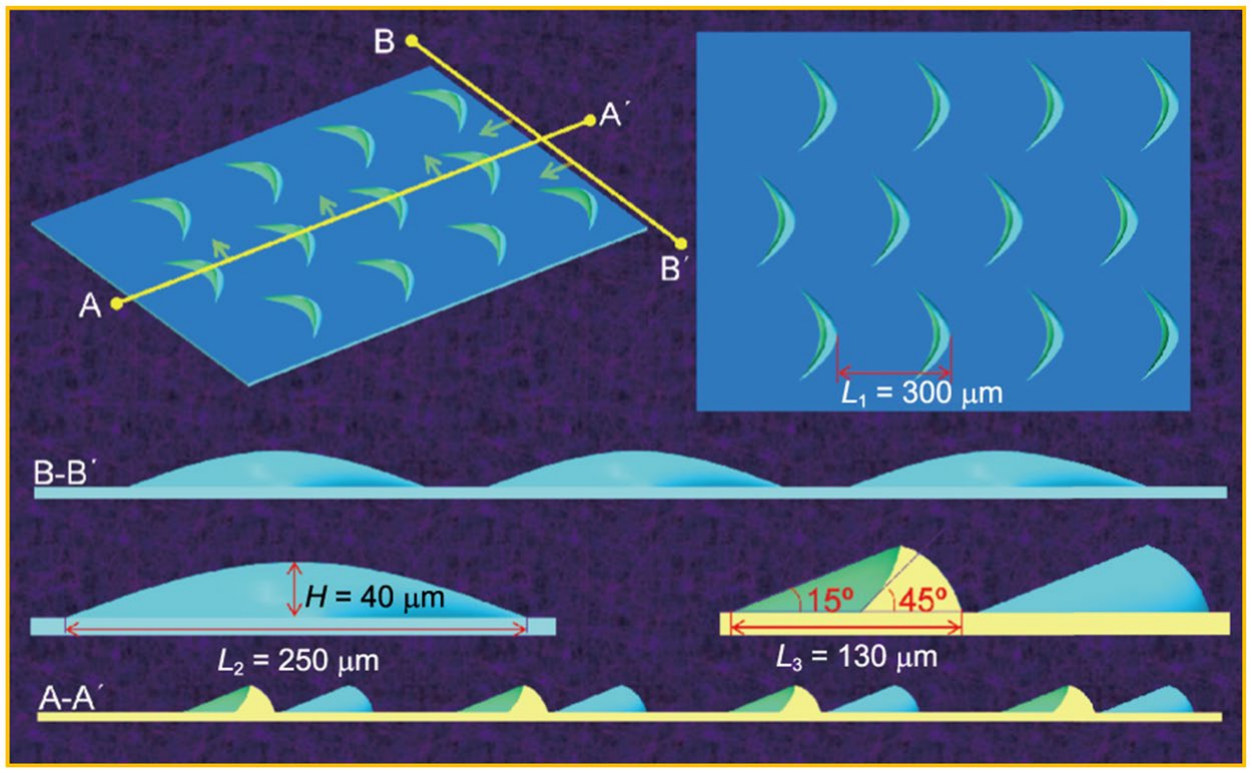
3D simplified model of the crescent-like ridge. It was used for numerical simulation. The space between neighbouring ridges is approximately *L*_1_ = 300 um. The width, length, and height of a single ridge is approximately *L*_2_ = 250 um, *L*_3_ = 130 um, and *H* = 40 um, respectively. Reprinted with permission from ref.^39^, Copyright 2017, Springer Nature.

Therefore, the peculiar functional structures of organism surfaces provide inspiration for solving key problems. According to the previous study^39^, the surface microstructures of the scales of the fish *Ctenopharyngodon idellus* were observed and analyzed. Then, the optimized 3D models that were designed based on the acquired data established. Finally, the mechanism of the water-trapping effect of these structures was analyzed through the numerical simulation. It was found that the crescent-like microstructures on the area of apical of the scales indicated remarkable drag-reduction effect, as shown in Fig. 1. A low-speed vortex was formed behind the crescent-like unit, and it effectively prevented the low-speed fluid moving out of the boundary layer. In fact, these crescents-like microstructures were effective in generating the “water-trapping” effect and forming a fluid-lubrication film, which reduced the skin friction drag effectively.

In this work, based on the basic principles of bionic research and previous numerical simulation results, morphological features of microstructure of fish scales has been simplified and a bionic surface with microstructures was fabricated to investigate the surface drag-reduction effect. The resistances of smooth and bionic surfaces experimental samples at different speeds were carefully investigated by the testing system for operation resistance. The contact angle (CA) of the surface of fish scales was researched by means of the contact angle measuring instrument to explore the wetting properties of the scales, which was used to further investigate the drag-reduction mechanism. Meanwhile, the analysis of the experimental results compared with the simulated ones was carried out. This work can have a great potential in wide sphere of engineering and transportation, and so forth for drag-reduction research at a low-speed environment.

## Experimental

### Resistance experiment

#### Experimental equipment

**Figure 2:**
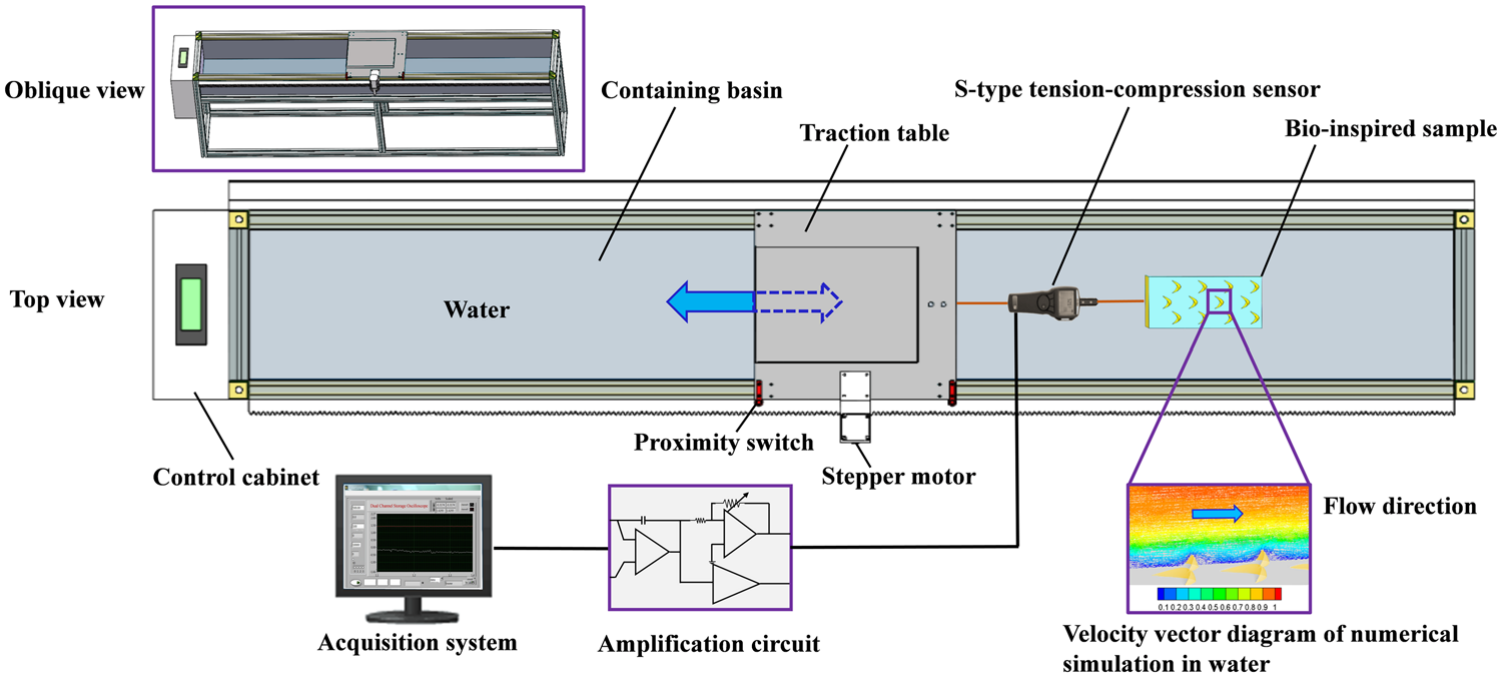
Schematic diagram of small experiment device on resistance.

Figure 2 shows the schematic diagram of the resistance-testing device, which is composed of a platform, drive system and measurement and control system. The testing device ensures that the experimental samples moving uniformly and is suspended by controlling the platform and limiting components of both ends of the device. The length, width and height of the bench were 2500 mm, 400 mm, and 920 mm, respectively. The length, width and height of the containing basin were 2400 mm, 320 mm and 200 mm, respectively.

As shown in Fig. 2, the dynamical system of gear rack was used to drive the transmission power. The rotational speed of the step motor (56BYG250DK) was controlled by the rotary encoder (E30S4-1000-3-N-24) that was coaxial with the step motor to make the traction table move back and forth at different speeds, which could maintain the continuous change speeds of the traction table between 0.1 and 1.0 ms^−1^. It was also clear that the test system consisted of the S-type tension-compression sensor (QLLY) and amplification circuit including BSQ-2 transmitting instrument, 24 V DC power and LabJackU12-type data acquisition card, as well as acquisition system. The range and power supply of the sensor was 0~50 kg and DC 24 V, respectively, which had the characteristics of high measurement precision and great stability. Consequently, the tension and compression force could be represented by the voltage signal, which was converted into force value through the calculation. In addition, the control system consisted of the core circuit in the control cabinet and control panel. And the control of normal- or abnormal-reverse twirl of step motor was composed of PLC, control panel, switch, wire, rotary encoder and step motor driver. The pulse instruction issued by PLC transferred to the step motor drive that controlled the rotary encoder and rotate speed of the step motor to achieve traction table’s movement and its variation in speed during the experiment.

#### Experimental model

**Figure 3:**
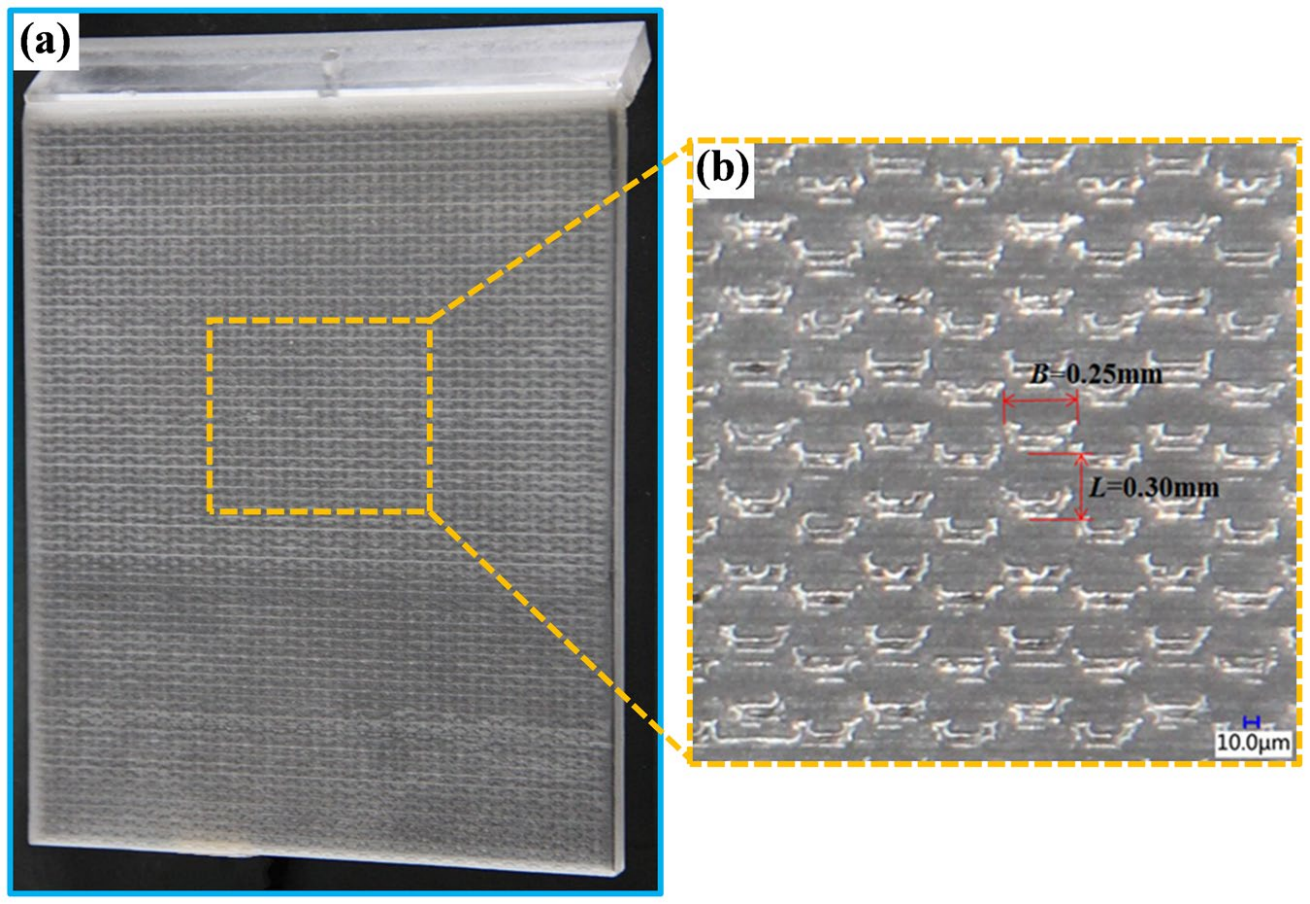
Experimental sample. (**a**) The experimental sample with delicate water-trapping microstructures of the fish *Ctenopharyngodon idellus* scales; (**b**) low-magnification sample; space between neighbouring ridges is approximately *L* = 300 μm, the width of a single ridge is approximately *B* = 250 μm.

By observing the microstructure of the scales, the main characteristic parameters were extracted intensively, that is, the space between neighbouring ridges was approximately *L*_1_ = 300 um, and the width, length, and height of a single ridge was approximately *L*_2_ = 250 um, *L*_3_ = 130 um, and *H* = 40 um, respectively. Next, the laser engraving machine (LENXAN) and polishing machine were used to fabricate the bionic experimental samples. Consideration must be given to the influence of the processing quality and weight of the experimental samples on the experimental results, finally, the thick acrylic hard plastic sheet about 10 mm was selected as the carrier of the experimental models. Figure 3 shows the experimental samples.

#### Working principle of the testing device

Firstly, the S-type tension-compression sensor was fixed in the rear of the traction table and connected with the input terminal of the BSQ-2 transmitting instrument. And the output terminal of the sensor connected with the positive and negative electrode of 24 V DC power. Secondly, the positive and negative electrode of the DC power connected with the positive and negative electrode of the LabJackU12-type data acquisition card, respectively. Also, the data acquisition card was connected to the computer via USB port to transmit the signals collected from the testing device. Finally, the experimental bio-inspired sample connected with the sensor through the thin wire rope of which the deformation could be ignored. The connection method of the testing device is shown in Fig. 2.

Next, the containing basin was cleaned up carefully in the pre-experiment and a correct amount of water was poured into it. The water height was approximate 40 mm. In addition, the data acquisition times and sampling frequency was 5 s and 500 Hz, respectively. In order to reduce the error of the experimental results, the data acquisition was commenced from 1039, excluding instability points of the experimental samples. In other words, the data of the same period are selected for calculation, and then, the average of 1000 experimental points that were converted to the force value were calculated and analyzed.

In this study, the microstructures of fish *Ctenopharyngodon idellus* scales were selected as biology prototype to explore the drag-reduction effect. With the aid of the bionic related theories, the life characteristics of five live fish (with average body length of approximately 39.6 cm, average body width of approximately 5.7 cm, and average body height of approximately 8.4 cm) were firstly analyzed in water. And a high-speed video camera (Dimax HD, PCO) was used to investigate the swimming speed in the water. It was observed that the average speed of the fish during the swimming process reached about 65.62 cm/s, which was consistent with the relevant data (65 cm/s), indicating that the fish was suitable for low-speed moving. Then in the experiment design, we first took the initial velocity of the fish as a reference, and thus the initial velocity was chosen to be 0.66 m/s. Meanwhile, it was found that as the flow speed increased, the drag reduction rate showed a downward trend by using numerical simulation method at different speed ranges (0.66–0.74 m/s) in our previous study^39^. Therefore, the flow-speed range was designed in the 0.66–0.74 m/s. The drag-reduction performance of the bionic surface was evaluated, and the drag-reduction rate was also calculated, as in Eq. (1).

1 $$\eta =\frac{{F}_{1}-{F}_{2}}{{F}_{1}}\times 100 \% $$

where *F*_1_ and *F*_2_ are the total drag of the smooth surface and bionic surface, respectively.

### Experiment of the CA measurement

#### Preparation of the materials

The scales, which were obtained from the area between the dorsal and ventral fins, of the fish *Ctenopharyngodon idellus* were selected as the observed samples. Firstly, the samples were immersed and washed in a 5%-concentrated sodium hydroxide (NaOH) solution for 3–4 h in order to remove excess mucus. Afterwards, the samples were cleansed using the KQ-100 medical ultrasonic cleaning device at 20 °C for about 1 h. Next, the samples were immersed in distilled water for 1~2 h and then fixed on the glass slide using double-sided tape for observing.

It was stated that the fish (*Ctenopharyngodon idellus*) scale samples were employed for carrying out the measurements of the contact angle and doing Scanning Electron Microscope (SEM) in this study; and Dr Liyan Wu was responsible for this and obtained a permission from his Shenyang Agriculture University. During the whole study, all the scale samples were bought from supermarket or peddlers. The fish were killed by normal technique and tools. There were no any kinds of live autopsy and cruel abuse. In addition, all fish treatments were definitely complied with the Chinese law on the Protection of Animals. Ethical approval was given by the animal Experimental Ethical Inspection, Shenyang Agricultural University.

#### CA measurement

The CA of the fish scales were measured by an optical contact angle measurement based on a sessile drop technique (OCA20 data physics, Germany), and the distilled water was used in the process of the experiment. The droplet volume is 7 μL. Because of different sizes of the microstructures distributing on the area of apical of fish scales, the same three groups that contained three measurement points for each group of the experiments were concurrently implemented to attain the relatively accurate results. Therefore, the average results of the multiple measurements gave the true value of CA in order to effectively decrease the deviation.

## Results and Discussion

### Resistance experimental results and analysis

Table 1 lists the electrical signal and sliding resistance values of the ordinary sample and bionic sample in the experiment, respectively. It was clearly seen that the electric signal value of the ordinary sample was larger than that of the bionic sample at the same flow speeds. Moreover, with the increase in flow speed, both two electric signal values were increasing, yet the signal values of ordinary sample were ultimate always larger than that of bionic sample.

**Table 1:** The measurement of electrical signals and sliding resistances of ordinary sample and bionic sample.

Velocity magnitude (ms^−1^)	Ordinary sample		Bionic sample	
	electrical signals (V)	sliding resistance (N)	electrical signals (V)	sliding resistance (N)
0.66	0.004129	0.020644	0.004013	0.020065
0.68	0.004188	0.020939	0.004074	0.020372
0.70	0.004193	0.020964	0.004080	0.020402
0.72	0.004223	0.021114	0.004111	0.020555
0.74	0.004276	0.021379	0.004165	0.020825

Here, the tension value of the sensor was linearly dependent with the output signal, which satisfied the following relation, as in Eq. (2).

2 $$U=\frac{1}{5}\times F$$

where *U* is the output signal of the sensor voltage (V); *F* is the drag force of the sensor (N).

By substituting the electrical signal in Table 1 into the Eq. (2), therefore, the corresponding resistance values of the bottom surface of bionic sample at various flow speeds could be obtained. It could be seen from Table 1 that the resistance value of the ordinary sample was larger than that of bionic sample at the same flow speeds. Furthermore, with the increase in flow speed, the resistance values of the ordinary sample and bionic sample were increasing, while the resistance of the former one was always greater than the latter one.

**Figure 4:**
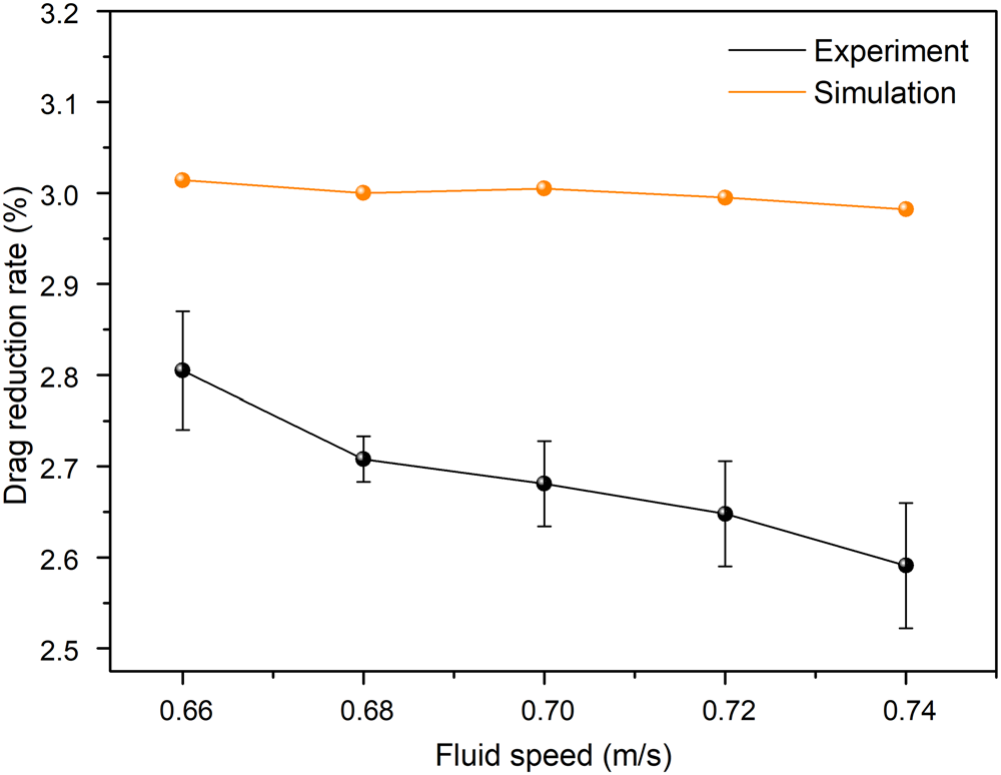
Comparisons of the drag reduction rate between experimental results and numerical simulation results at different flow speeds.

Figure 4 illustrates the comparisons of the drag-reduction rate between the experimental and numerical simulation results at different flow speeds. As seen from Fig. 4, with an increase in the incoming flow velocity, the drag-reduction rate of the experimental and simulated results simultaneously displayed a downward trend in the entire speed span (0.66–0.74 m/s). As a result, the maximum drag-reduction rate of the experimental and simulated results was 2.805% and 3.014%, respectively, when the flow rate was approximately 0.66 m/s. In particularly, the calculation result showed that the error of the drag reduction rate was 0.085% at the speed of 0.66 m/s, which was less than the drag-reduction rate of 2.856% obtained by the experiment. Furthermore, it was obvious that the experimental results were a little lower than that of the simulated one, but both two were in the allowed error tolerance range. The possible causes of this phenomenon were as follows: on the one hand, combining with some characteristics of numerical simulation, a three-dimensional model of “crescent-like” microstructures with 12 rows was used in numerical simulation. In the actual experiment, however, the surface microstructures of the bioinspired sample were several hundred times as many as the surface of the model in numerical simulation. On the other hand, the machining accuracy of the crescent-like microstructures on the surface of the bioinspired sample may not matching that of the model employed in numerical simulation, resulting in an increase in contact areas between the water and the top of bioinspired sample. In short, the numerical simulation and experimental results indicated that the bioinspired surface possessed a good drag-reduction effect, and the error between them had a range of 6.934~13.112%. It could be accepted that the drag-reduction effect of the numerical simulation and experiment was approximate to each other.

Furthermore, the results further showed that the bionic surface with water-trapping microstructures of fish scales had a great drag-reduction effect at low-speed fluid environments. In the previous numerical simulation work, the drag-reduction characteristic of bionic surface with delicate water-trapping microstructures had been researched and detailedly revealed drag-reduction mechanism^39^. Therefore, the experimental results were conformation of the numerical simulation results on the drag-reduction rate, which further verified the drag-reduction characteristics of the bionic surface with water-trapping microstructures.

### Experimental results and analysis of the CA measurement

**Figure 5:**
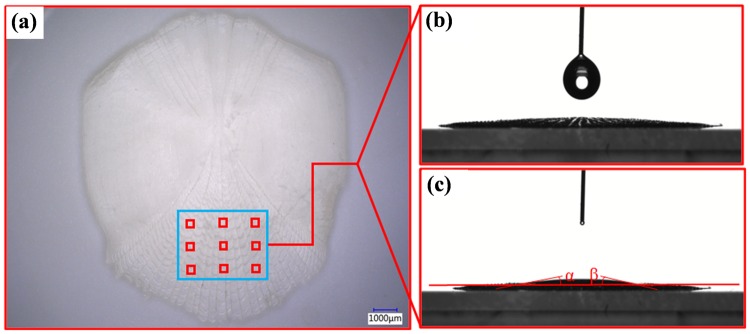
The image of the CA of fish scales surface. (**a**) The schematic diagram of CA measurement points; (**b**), (**c**) the images of the CA of fish scales surface.

Figure 5(a) shows the schematic diagram of the CA measurement points of fish scales. The measurement results of surface wetting angles are given in Fig. 5(b,c). It was seen from the results that the left and right CA of the scales was not totally equal. Namely, the *α* and *β* was 10° and 13°, respectively. Consequently, the average values of the left and right CA of the scales were taken to represent the final CA measurements of the scales surface in this experiment. It could be clearly seen that the CA was about 11.5° by calculation, which showed remarkable hydrophilic properties. It further demonstrated the spreading-wetting phenomenon and the higher surface energy for the area of apical of fish scales.

In fact, for the rough surfaces, the Wenzel model suggested that the droplets would be completely immersed into the microstructures of the rough surface, thus forming the wetting contact of the non-compound state. The equation is following^40,41^:

3 $$\cos \,{\theta }_{w}=r\,\cos \,{\theta }_{Y}$$

where *θ*_*w*_ is the apparent contact angle of the Wenzel model, *r* represents the roughness of the solid surface ($r={S}_{a}/{S}_{{\rm{g}}}$, *S*_a_ and *S*_g_ is the actual surface area and apparent surface area, respectively), *θ*_*Y*_ stands for the eigen contact angle.

**Figure 6:**
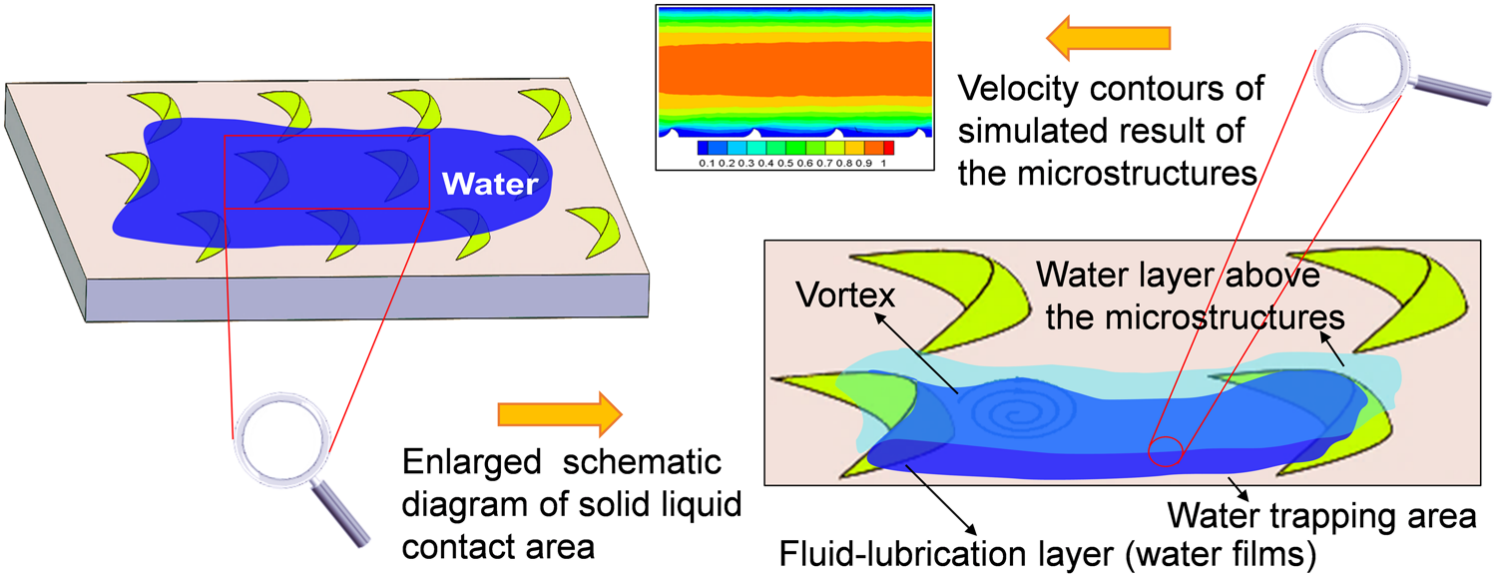
Schematic illustration of the water-trapping effect among crescent-like microstructures, showing a hydrophilic wetting state.

It also could be clearly seen, from Fig. 5(b,c), that the droplet spread quickly when it dripped on the microstructure surface, which generated the wetting contact of the non-compound state on the scales surface. It was noted that the mobility of the droplet slowed down dramatically between the adjacent crescent-like microstructures, even produced an adhesive effect on the surface. One of the main reasons for this phenomenon might be resulted from the crescent-like microstructures distributing on the area of apical, which played a positive role in creating a high interface energy surface. Thus, the surface property of fish scales was changed, showing hydrophilic properties. This directly supported that the water-trapping areas between adjacent microstructures could be well reserved liquids (mucus, water, or other mediums) by the interaction between the areas and liquids, which contributed to reduce the frictional resistance of the fish scale surfaces. Figure 6 indicates the schematic illustrations of the water-trapping effect among crescent-like microstructures, showing a hydrophilic wetting state. As shown, a low-speed fluid gathered in the neighboring crescent-like microstructures generated a stable water-trapping area. And the vortex of a low-speed area was generated behind the crescent-like unit; this either effectively prevented the external high-speed fluid from moving into the area or the low-speed fluid from moving out of the boundary layer. This property could form a stable “reserve liquid” effect and avoid mucus reduction. Importantly, the spreading wettability of liquids in this area might be regarded as a fluid-lubrication layer, which formed a contact between water and water instead of the scale material. And all stable low-speed water areas joined together because of the distribution of all crescents-like units. Therefore, the self-formed fluid-lubrication film could produce a drag-reduction performance^39^. With respect to the as-received fish scales, however, these microstructures also could originally avoid excessive loss of mucus, and then the drag-reduction was realized.

## Conclusions

In summary, the resistances of smooth and bionic surfaces experimental samples at different speeds were carefully investigated by the testing system, and the contact angle (CA) of fish *Ctenopharyngodon idellus* scales surface was measured by means of the contact angle measuring instrument. At first, it was found that the bionic surface with water-trapping microstructures dictated a remarkable drag-reduction effect at a low speed, and the drag-reduction rate significantly displayed a downward trend with the increase in flow speed. In contrast to the smooth surface experimental sample, when the rate was 0.66 m/s, the drag-reduction effect of the bionic surface was the best, the maximum drag reduction rate was 2.805%, which was in concordance with the simulated one. In summary, the experiment was conformation of the numerical simulation results on the drag-reduction rate and verifies the drag-reduction characteristics of the bionic surface. When the surface of the contact angle (CA) was about 11.5°, it showed remarkably hydrophilic properties. It further demonstrated the spreading-wetting phenomenon and the higher surface energy for the area of apical of fish scales. Actually, the crescent-like microstructures distributing on the area of apical, to a certain extent, primarily played a positive role in changing the surface properties of fish scales. As a result, the spreading wettability of droplets in this area might be regarded as a fluid-lubrication layer, which formed a contact between water and water instead of the scale material, to reduce the fluid resistance. This work can have great potential in the engineering, transportation field and drag-reduction research at a low-speed environment.
